# A comparison of Y-chromosomal lineage dating using either resequencing or Y-SNP plus Y-STR genotyping^☆^

**DOI:** 10.1016/j.fsigen.2013.03.014

**Published:** 2013-12

**Authors:** Wei Wei, Qasim Ayub, Yali Xue, Chris Tyler-Smith

**Affiliations:** The Wellcome Trust Sanger Institute, Wellcome Trust Genome Campus, Hinxton, Cambridgeshire CB10 1SA, UK

**Keywords:** Human Y chromosome, Male history, Time estimation, Networks, BATWING

## Abstract

We have compared phylogenies and time estimates for Y-chromosomal lineages based on resequencing ∼9 Mb of DNA and applying the program GENETREE to similar analyses based on the more standard approach of genotyping 26 Y-SNPs plus 21 Y-STRs and applying the programs NETWORK and BATWING. We find that deep phylogenetic structure is not adequately reconstructed after Y-SNP plus Y-STR genotyping, and that times estimated using observed Y-STR mutation rates are several-fold too recent. In contrast, an evolutionary mutation rate gives times that are more similar to the resequencing data. In principle, systematic comparisons of this kind can in future studies be used to identify the combinations of Y-SNP and Y-STR markers, and time estimation methodologies, that correspond best to resequencing data.

## 1 Introduction

The combination of its male-specific inheritance, small effective population size and geographical specificity make the Y chromosome the locus of choice for investigating many questions about both forensics [1] and human male history and prehistory [2]. In some cases, the distribution [3] or sharing [4,5] of a Y-chromosomal lineage may itself provide the information sought, but often an estimate of a date or time is an integral part of a study: for example, the time when a lineage originated [6–8] or spread [9], or when a population began to expand in numbers [10,11]. Such a date can then be compared with other genetic or non-genetic dates to generate integrated insights.

Two kinds of data are needed in order to obtain a date estimate from present-day Y chromosomes: information about the genetic diversity of the Y chromosomes, and a measure of the mutation rate of the loci used to determine the diversity. Over the last two decades, Y-chromosomal studies have increasingly used Y-STRs to measure genetic diversity [2]. The commonly used Y-STRs are variable in all populations (http://www.yhrd.org/) and mutate quickly enough that their mutation rates can be measured in deep-rooting families [12] or father–son pairs [13], and refined using information from levels of population variation [14]. Thus Y-STR mutation rates are now estimated with some precision. Nevertheless, there are complications in using variants that mutate so fast (about once in 500 transmissions) to estimate evolutionary times: after ∼15 thousand years, a typical Y-STR will have mutated more than once. Since most mutations are increases or decreases of single repeat units [13], about half of double mutations will recreate the original allele and thus not be readily detected by simply examining the haplotype. A number of approaches have been taken to address such issues. The construction of networks representing the evolutionary history of a set of haplotypes can recover some of the non-observed haplotypes [15], and this process is aided by including Y-SNPs (which have much lower mutation rates) in the network, or analysing only single groups of closely related haplotypes defined by Y-SNPs (haplogroups). Times can be estimated from networks of such data using the rho statistic [16]. Other approaches to time estimation include models of the Y-STR mutational process that allow back-and-forth mutations [17]. Nevertheless, it has also been suggested that mutation rates measured in families or father-son pairs should be calibrated to lower values when used for evolutionary purposes [18,19].

While these methods for estimating times have found widespread acceptance and use, it has been difficult to assess their reliability because of the lack of datasets where the true time of interest is known. Indeed, comparisons with simulated datasets have suggested that times estimated from networks using the rho statistic are not always reliable [20]. Improvements in technology now mean that another form of test, comparison with Y-chromosomal variation discovered by large-scale resequencing, is possible [21]. Such comparisons have the advantage that the variation discovered by resequencing and Y-STR genotyping on a lineage share the same history, so testing of reliability is not complicated by, for example, choice of a particular demographic scenario in a simulation. In addition, Y-SNPs, the most abundant variants discovered by resequencing, mutate so slowly that recurrent mutations on the human Y chromosome will generally have negligible effects on resequencing-based time estimates.

We have previously constructed a calibrated phylogenetic tree based on Y-SNPs discovered by resequencing ∼9 Mb of unique Y-chromosomal DNA, or subsets of this DNA [21]. The branch lengths (numbers of SNPs) on this tree between any pair of chromosomes are proportional to the time separating the chromosomes, and since these SNP numbers are large, usually a few hundred, they are determined accurately. However, since substantial effort is required to generate ∼9 Mb of sequence data per individual and the number of Y chromosome sequences available is still small, we wished to compare conclusions from full resequencing with conclusions from more standard Y-STR plus Y-SNP genotyping, where vastly more datasets are available. We have therefore typed this set of resequenced Y chromosomes with 23 common Y-STRs, applied a widely used method for estimating times from traditional Y-SNP plus Y-STR genotypes, and compared the time estimates with those from resequencing.

## 2 Materials and methods

### 2.1 DNA samples and genotyping

We analysed 33 of the 36 males for whom sequence data from the Y chromosome are available [21]; the three individuals not included in the current study were three of the sons in the CEU pedigree. 32 of these individuals had been sequenced by Complete Genomics, and one by The Wellcome Trust Sanger Institute, as reported in the previous study [21]. DNA samples were obtained from the Coriell Institute for Medical Research (Camden, NJ, USA).

23 Y-STRs were genotyped using the PowerPlex^®^ Y23 system (Promega). Each sample was amplified in 5 μl volume containing 1 μl of PowerPlex^®^ Y23 5X Master Mix, 0.5 μl of PowerPlex^®^ Y23 10X Primer Pair Mix and at least 0.5 ng of template. Cycling conditions used an initial denaturation of 96 °C for 2 min, 30 cycles of 94 °C for 10 s, 61 °C for 1 min, and 30 s at 72 °C followed by a 20 min hold at 60 °C and a final 4 °C soak using the max ramp rate on a MJ Research DNA Engine Tetrad 2. Separation of amplification products was performed on the Applied Biosystems 3730xl. A 36 cm capillary array was used with POP-7^®^ Polymer (Life Technologies™). Samples were prepared for separation and analysis by adding 1 μl of 1:10 dilution amplified sample or allelic ladder to 10 μl of Hi-Di™ Formamide (Life Technologies™) and 1 μl of CC5 Internal Lane Standard 500 (ILS). Samples were denatured for 3 min at 95 °C followed by a snap cool in an ice bath and subsequently injected for 23 s at 1.2 kV. GeneMapper^®^ ID Software, Version 3.0 (Life Technologies™) was used to determine fragment size and allele calls with a 50 RFU analytical threshold. Genotypes for a set of 29 standard Y-SNPs that define the major Y haplogroups (Supplementary Table 1) were extracted from the sequence data.

Supplementary Table 1 Y-SNP and Y-STR genotypes in the 33 samples analysed. The yellow highlights indicate Y-STRs that show a mutation within the family.

### 2.2 Data analysis

Median-joining networks [15] of haplotypes consisting of 21 Y-STRs and 29 Y-SNPs were constructed using Network 4.61.1 (http://www.fluxus-engineering.com/sharenet.htm). The duplicated locus *DYS385* was not used in these analyses since the constituent loci are not distinguished in this assay, and *DYS389* was treated as *DYS389I* and *DYS389b* [*DYS389b* = *DYS389II* − *DYS389I*]. The 29 Y-SNPs were assigned high weights of 99, and the 21 Y-STRs lower weights that ranged from 1 to 5, depending upon the inverse of the variance of each STR (Supplementary Table 1). We manually counted the number of Y-STR mutational steps on the network between each pair of individuals.

Time estimates were made using BATWING (Bayesian Analysis of Trees With Internal Node Generation) [17] based on 26 Y-SNPs and 21 Y-STRs. We excluded three SNPs that were not variable in the 33 individuals, treated *DYS385* and *DYS389* as above, and used a population model of exponential growth from an initially constant-sized population with the settings, priors and convergence assessments described previously [10]. Five sets of Y-STR mutation rates were used. These included two compilations of “observed” mutation rates (OMR) [11,14], a widely used calibrated “evolutionary” mutation rate (EMR) [19], a recalibrated evolutionary mutation rate (rEMR) [11] and a mutation rate predicted from the logistic model (lmMR) [14] (Supplementary Table 2). We evaluated both the Time to the Most Recent Common Ancestor (TMRCA) of the entire sample, and the times of individual Y-SNPs within the phylogeny. The time estimates for individual Y-SNPs were compared with the times of the same SNPs estimated by GENETREE from sequence data, as reported previously [21]. Pearson's correlation coefficient (*R*^2^), Spearman's rank correlation coefficient (rho), and their significance were calculated using the correlation test in R2.15.1 (http://www.r-project.org).

Supplementary Table 2 Five sets of Y-STR mutation rates and the prior distributions used in BATWING analyses.

## 3 Results

**Fig. 1:**
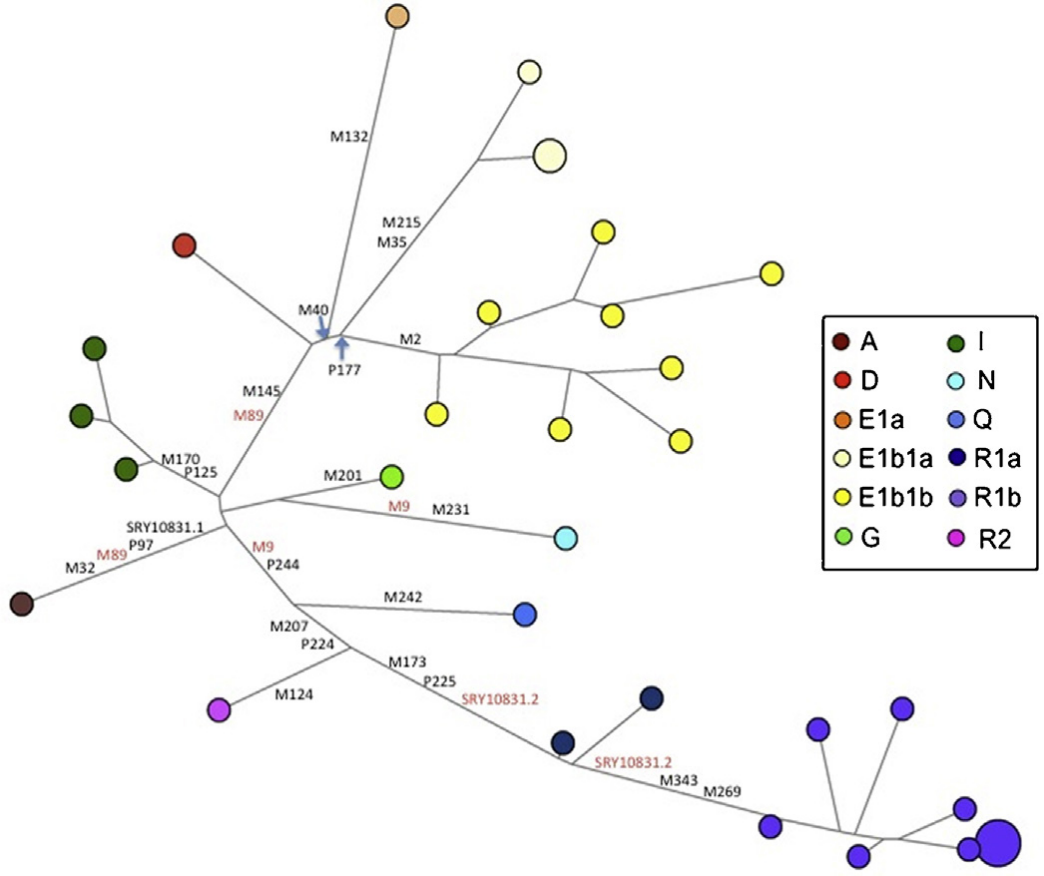
Median-joining network representing the relationships between 33 Y chromosomes based on 26 variable Y-SNPs and 21 Y-STRs. Each circle represents a haplotype and has an area proportional to its frequency. All haplotypes are present once, except one within E1b1a and one R1b. Lines represent Y-SNP plus Y-STR mutational steps between the haplotypes and have a length proportional to the number of steps. The branches on which the Y-SNP mutations lie are indicated; note that there is no information about location or ordering within the branch on which they lie. Y-SNP names in red represent sites assigned as recurrent on the network but not on the sequence-based phylogenetic tree in Supplementary Figure 1.

We generated a standard: Y-SNP plus Y-STR dataset for 33 of the 36 Y chromosomes previously analysed by resequencing. The 29 Y-SNPs were chosen to correspond to those that might have been used if the resequencing data had not been available: they defined common haplogroups (including three that were not present in this sample) and subdivided haplogroups known to be frequent in Africa and Europe, where many of the samples originated. The 23 Y-STRs included those most commonly used. We began by constructing a phylogenetic network from the data (Fig. 1). The resulting network clusters groups of chromosomes from the same haplogroup together and reconstructs most of the expected features of the phylogeny. For example, the diverse E1b1b chromosomes are grouped together, next to their sister group E1b1a, with the two E1b haplogroups linked next to E1a and then D. However, not all deep relationships are reconstructed correctly. R1a chromosomes are placed between R2 and R1b, with the SRY10831.2 SNP mutation that defines this haplogroup being incorrectly assigned as recurrent, mutating from G to A between R2 and R1a, and then back from A to G between R1a and R1b. Similarly, the M89 and M9 mutations deep within the network are placed as recurrent, showing that ancient structure is not reconstructed correctly here.

**Supplementary Figure 1:**
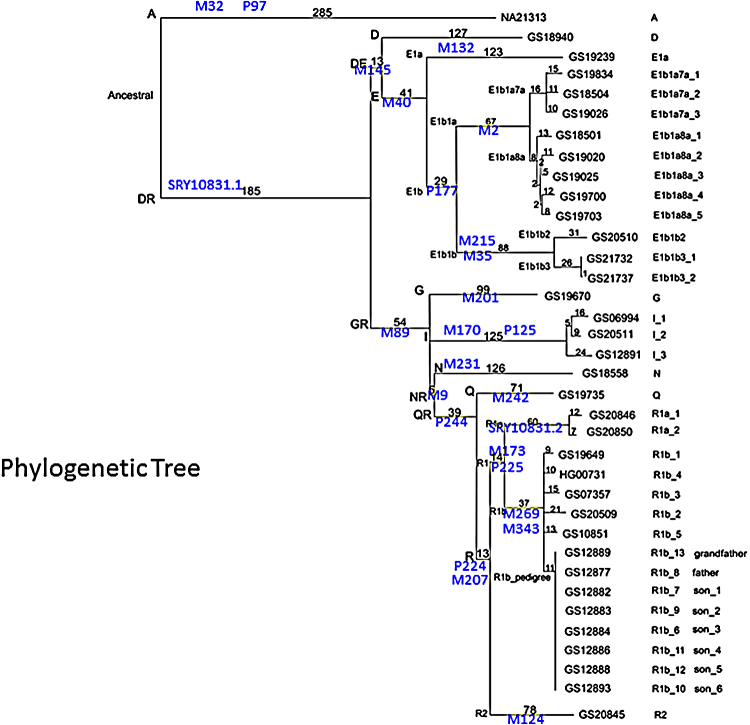
Phylogenetic tree based on resequencing [21] showing the locations of the 26 Y-SNP mutations used in the current study.

**Fig. 2:**
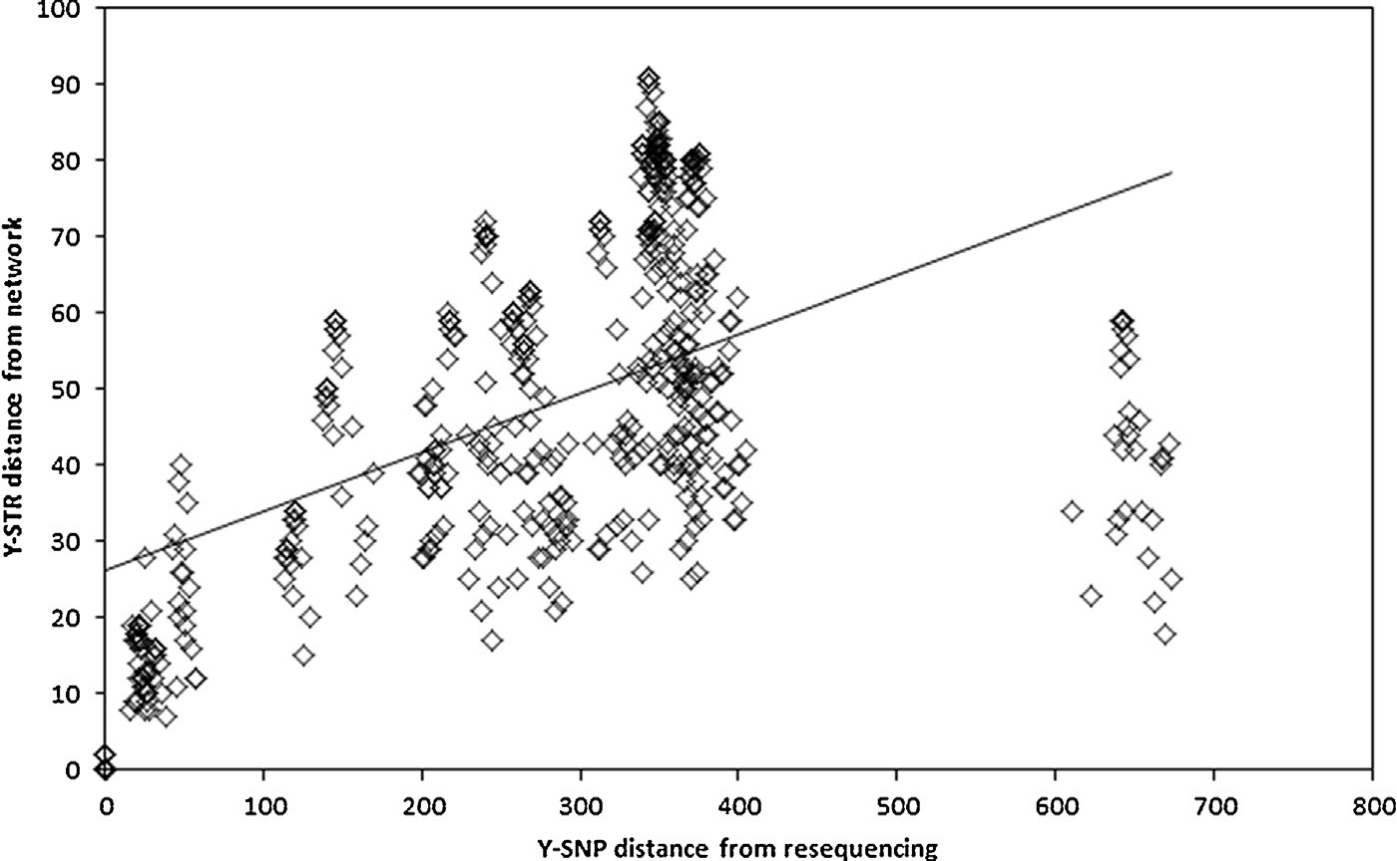
Comparison of Y-SNP and Y-STR distances between pairs of chromosomes. Y-SNP distances were determined from the phylogenetic tree based on resequencing, while the Y-STR distances were determined from the Y-SNP plus Y-STR network shown in Fig. 1.

We next compared the Y-SNP distance on the previous resequencing-based phylogenetic tree (Supplementary Figure 1) between each pair of chromosomes with the Y-STR distance on the network between the same pair of chromosomes (Fig. 2). Although the absolute numbers will differ, they should be correlated. They are indeed very significantly correlated (*p* < 2.2 × 10^−16^), but the value of the Spearman correlation coefficient is only 0.52 and Pearson's *R*^2^ only 0.28, reflecting both the large scatter of points seen in Fig. 2 and the striking saturation of Y-STR distances for chromosomes separated by large Y-SNP distances (Fig. 2): on the sequence-based tree, the haplogroup A chromosome is very distinct from all of the others (Supplementary Figure 1), but on the network it does not lie on an exceptionally long branch (Fig. 1). These findings also alert us to the possibility that conclusions about deep relationships based on genotyping Y-SNPs plus Y-STRs may be unreliable.

**Fig. 3:**
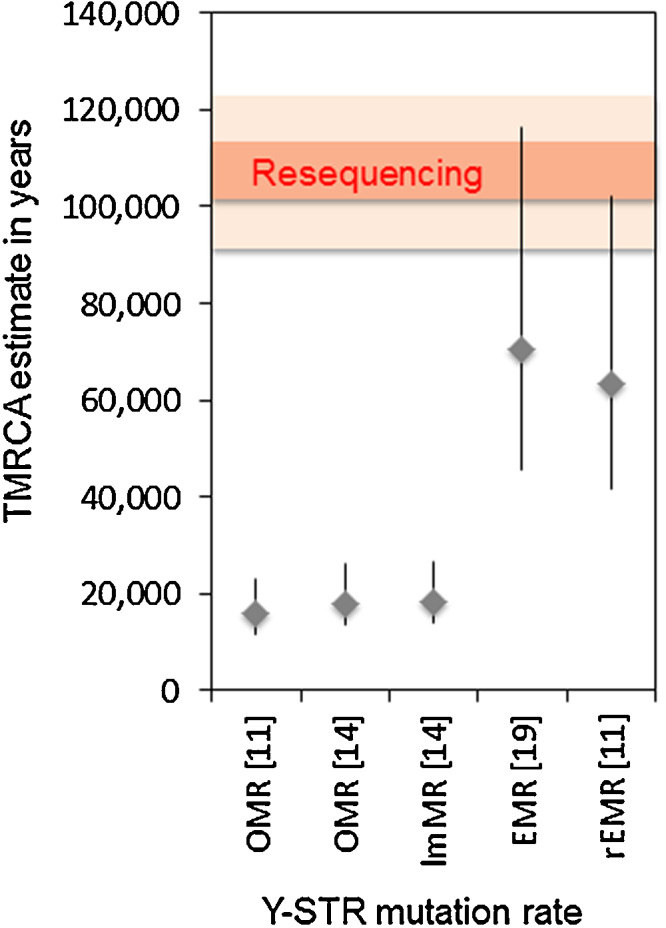
Comparison of TMRCA estimates based on Y-SNP plus Y-STR genotyping using five different Y-STR mutation rates, with the range of published estimates based on resequencing. Dark horizontal bar: range of five point estimates from resequencing; light horizontal bar: standard deviation of the point estimate with the largest uncertainty [21].

Distances measured in mutational steps can be converted into times measured in years if the mutation rate is known, for example using the program BATWING. While there have been numerous measurements of Y-STR mutation rates, there is, as discussed above, controversy about which rate should be used in evolutionary studies. We therefore compared times estimated from the resequencing data with times estimated using five different assessments of the Y-STR mutation rate: two compilations of observed mutation rates, an observed mutation rate adjusted for population variation, an evolutionary mutation rate and a recalibrated evolutionary mutation rate (Supplementary Table 2). For simplicity, we present here a single time estimate: that of the TMRCA of 33 chromosomes, based on each of the mutation rates. For the resequencing data, five point estimates made using the same dataset and calibration rate but different methods of calculation were available. These ranged from 101 to 115 KYA [21] (Fig. 3, shaded horizontal bar). In contrast, the TMRCAs estimated from the Y-SNP plus Y-STR data using the five different Y-STR mutation rates were all much younger and ranged from 16 to 71 KYA (Fig. 3). In this comparison, the TMRCAs based on the two evolutionary mutation rate estimates are more consistent with resequencing-based TMRCAs than those based on the observed mutation rates: the EMR, rEMR and resequencing confidence intervals overlap. The resequencing-based TMRCAs of course depend on the calibration used, in this case derived from a small number of observed Y-SNP mutations in a deep-rooting pedigree [22]. Nevertheless, the resequencing-based TMRCAs are consistent with well-supported dates such as the migration of modern humans out of Africa ∼60 KYA [23], while the much more recent dates estimated using the observed Y-STR mutation rates are highly implausible.

**Fig. 4:**
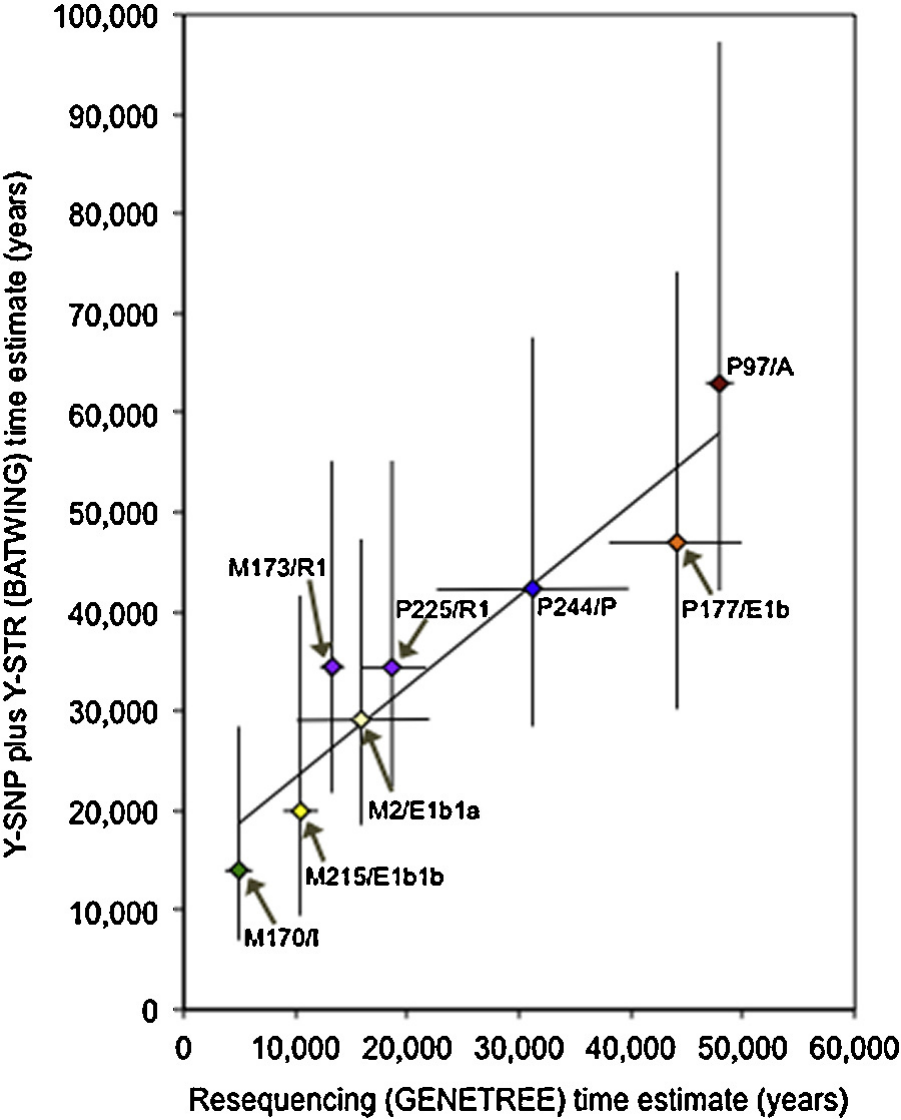
Comparison of time estimates from Y-SNP plus Y-STR genotyping with published time estimates based on resequencing for eight Y-SNPs within the phylogeny. Colours represent the haplogroups defined by the SNPs, using the same conventions as Fig. 1.

Since the EMR produced the time estimates most consistent with the resequencing-based estimates, we compared the times of additional nodes within the tree between these two methods (Fig. 4). The two sets of time estimates are highly correlated (Spearman's rho = 0.96, *R*^2^ = 0.88, *p*-value = 0.00018).

## 4 Discussion

In this study, we have documented large differences between conclusions about times based on full resequencing compared to standard Y-SNP plus Y-STR genotyping. Here, we discuss three major aspects of these differences, and the more general conclusions that may be drawn from our study.

First, and independent of any choices about calibration rate, Y-STR mutation counts between lineages appear to saturate rapidly (Fig. 2). A line relating the two must pass through the 0,0 point on the graph; if a straight line were drawn through this point based on the Y-SNP distances <100, under-counting of Y-STR steps would be apparent even for Y-SNP distances of 100–200. It is widely appreciated that raw Y-STR mutational differences between haplotypes saturate rapidly, but the use of networks, especially those incorporating Y-SNPs, is expected to recover many of the Y-STR mutational steps obscured by recurrent mutation. We see that this strategy is only partially successful.

Second, the comparison with time estimates based on resequencing provides an opportunity to evaluate the different Y-STR mutation rates that have been proposed. Even when times are estimated using an approach that models recurrent mutation [17], the use of observed mutation rates leads to time estimates that are several-fold too recent. In contrast, an evolutionary mutation rate [19] leads to more plausible time estimates. The Y-SNP mutation rate [22] used for calibration of the resequencing data has itself wide confidence intervals, so could these instead be responsible for the discrepancy? Current debate about the human SNP mutation rate and its implications for the timing of evolutionary events contemplates the possibility of a longer timescale rather than a shorter one [24], so this seems unlikely: older Y-sequence-based times would be even less consistent with the observed Y-STR mutation rates.

Third, if the Y-STR mutation rate that generates a TMRCA that matches the resequencing TMRCA is chosen (i.e. the EMR), the times of additional nodes in the tree also match. While this finding is expected, it is nevertheless reassuring to see the strength of the correlation between the resequencing times calculated by GENETREE and the Y-SNP plus Y-STR times calculated by BATWING.

Despite the limitations identified, we now have a tool to evaluate time estimates based on Y-SNP plus Y-STR genotyping in a systematic way. For example, do some Y-STRs lead to more reliable estimates than others [8], and can a subset of the most useful ones be identified? How does the inclusion of Y-SNPs influence the time estimates? Do alternative methods of estimating time from Y-SNP plus Y-STR data correspond more or less well with resequencing data? Do bigger datasets, especially ones containing groups of more closely related chromosomes, lead to better recovery of recurrent mutations and thus more reliable time estimates?

## 5 Conclusions

We have compared a laborious and expensive ‘gold standard’ method for estimating Y-chromosomal lineage times – resequencing ∼9 Mb of DNA – with the much easier and more cost-effective standard approach of genotyping sets of Y-SNPs plus Y-STRs. While the times estimated using the two approaches can vary several-fold, we conclude that BATWING time estimates based on an evolutionary mutation rate correlate best with the resequence data.
